# Reflections on the International Bioethics and Humanities Conference 2024

**DOI:** 10.51866/mol.920

**Published:** 2025-04-26

**Authors:** Hasliza Abu Hassan, Nurul Aida Fathya, Astrid Sinarti Hassan

**Affiliations:** 1 MBBS, MMed (Fam Med), Family Medicine Department, Universiti Pertahanan Nasional Malaysia, Kem Perdana Sungai Besi, Malaysia. Email: hasliza@upnm.edu.my; 2 MD, Forensic and Medicolegal Specialist, M.Sc (Bioethics), Department of Forensic and Medicolegal, General Achmad Yani University, Cimahi, West Java, Indonesia.; 3 MB BCh BAO, MMed Ethics and Juris, PhD, Department of Medical Ethics and Law, Faculty of Medicine, Sungai Buloh Campus, Universiti Teknologi MARA, Malaysia.

**Keywords:** Bioethics, Education, Interprofessional relations

## Introduction

Attending the International Bioethics and Humanities Conference (IBHC) 2024 in Yogyakarta, Indonesia, was an enriching experience that provided a platform to engage with global bioethics experts and discuss issues in medicine, technology and environmental sustainability.

It was my first time presenting a research project at a bioethics conference, marking an important milestone in my career. The event, held at Alana Hotel, began with a 1-day preconference on November 5, followed by the main conference from 6 to 8 November 2024, bringing together medical experts, and bioethicists to foster bioethics education and knowledge sharing.

## Preconference highlights

During the preconference, five themed workshops offered a platform for interdisciplinary discussions on diverse bioethics topics. Led by experts, these workshops explored decolonizing bioethics, ethics in traditional medicine, clinical ethics support, palliative care, and research ethics committees. Participants from diverse disciplines, including medicine, philosophy, law, biotechnology and environmental science engaged in critical discussions on the ethical challenges posed by emerging technologies, global health issues, and human rights. Integrating insights from various disciplines, these sessions enhanced participants' ethical analysis skills and preparing them for deeper discussions at the main conference.

## Key themes at the main conference

The IBHC 2024 symposiums addressed key bioethics issues, including biobanking ethics in Southeast Asia, focusing on informed consent, data privacy and equitable access to health innovation. Discussions on bioethics education emphasized interactive approaches to fostering ethical leadership in higher education. Precision medicine sessions explored data privacy, consent frameworks, and genomic access. Healthcare ethics discussions include palliative care, rare disease management, cultural influences on medical decisions, traditional medicine, research conduct, and the impact of local cultures on environmental sustainability. Keynote addresses, symposia, and panel discussions explored ethics in technology, climate change, and human dignity, while the experience was enriched by exploring the culture and beauty of Yogyakarta.

## My research presentation: Ethical issues in gift-giving

At IBHC 2024, I presented my poster entitled ‘Ethical Issues in Gift-Giving’ in healthcare, exploring conflicts between professionalism and personal relationships. The feedback and discussions enriched my research and broadened my perspectives. Engaging with the bioethics community reinforced the relevance of my research. Additionally, collaboration and mentorship from lecturers at Universiti Teknologi MARA deepened my understanding of medical ethics, enhancing my research approach and commitment to advancing ethical studies in healthcare.

I also met Dr Nurul Aida Fathya, a bioethicist from Universitas Jenderal Achmad Yani. Her research on interprofessional collaboration in Indonesia highlighted the shared values and ethical principles that enhance interprofessional performance. Our discussions led to online meetings exploring potential collaborations and grants, while the conference broadened my understanding of global bioethics and paved the way for future research collaborations.

The photographs taken during the event are attached in this article.

**Figure 1:**
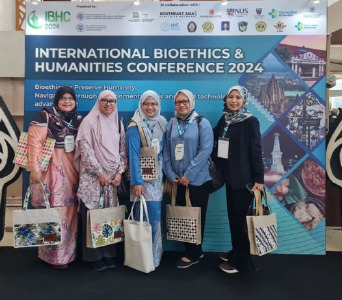
With delegates from Malaysia, UPNM, Universiti Teknologi MARA and Universiti Sains Islam Malaysia.

**Figure 2:**
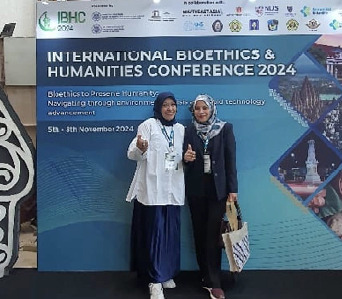
With Dr Nurul Aida Fathya, a forensic specialist and a bioethicist from Universitas Jenderal Achmad Yani.

**Figure 3:**
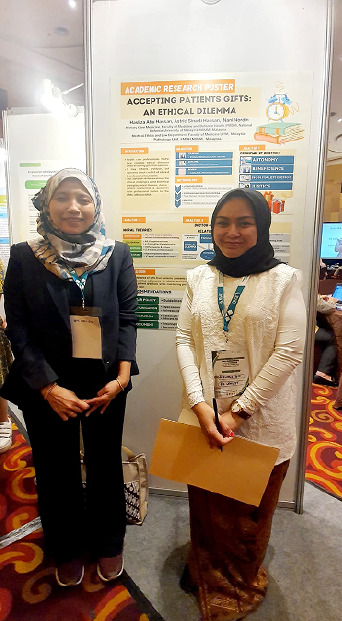
Presentation of my poster entitled 'Ethical Issues in Gift-Giving'.

**Figure 4:**
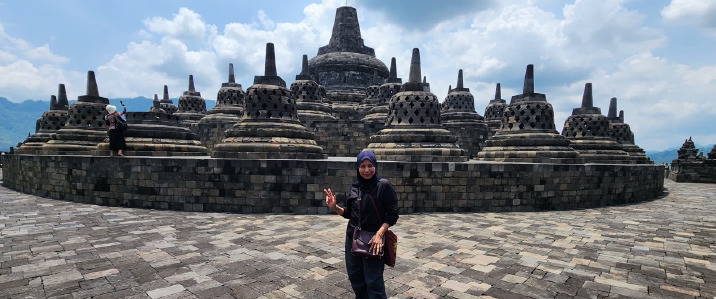
A visit to the historical Borobudur Temple.

## Conclusion

Participating in the IBHC 2024 was a transformative experience, reinforcing my passion for bioethics. It gave me the confidence to continue presenting my research at international platforms and explore ethical dilemmas in medicine and technology. This conference was a stepping stone in my journey, and I look forward to more opportunities for meaningful discussions and collaborations in the field of bioethics.

